# The Movement- and Load-Dependent Differences in the EMG Patterns of the Human Arm Muscles during Two-Joint Movements (A Preliminary Study)

**DOI:** 10.3389/fphys.2016.00218

**Published:** 2016-06-08

**Authors:** Tomasz Tomiak, Tetiana I. Abramovych, Andriy V. Gorkovenko, Inna V. Vereshchaka, Viktor S. Mishchenko, Marcin Dornowski, Alexander I. Kostyukov

**Affiliations:** ^1^Unit of the Theory of Sport and Motorics, Chair of Individual Sports, University of Physical Education and SportGdansk, Poland; ^2^Department of Movement Physiology, Bogomoletz Institute of Physiology, National Academy of SciencesKiev, Ukraine

**Keywords:** motor control, two-joint movements, muscle synergy, motor commands, electromyogram

## Abstract

Slow circular movements of the hand with a fixed wrist joint that were produced in a horizontal plane under visual guidance during conditions of action of the elastic load directed tangentially to the movement trajectory were studied. The positional dependencies of the averaged surface EMGs in the muscles of the elbow and shoulder joints were compared for four possible combinations in the directions of load and movements. The EMG intensities were largely correlated with the waves of the force moment computed for a corresponding joint in the framework of a simple geometrical model of the system: arm - experimental setup. At the same time, in some cases the averaged EMGs exit from the segments of the trajectory restricted by the *force moment singular points* (*FMSPs*), in which the moments exhibited altered signs. The EMG activities display clear differences for the eccentric and concentric zones of contraction that are separated by the *joint angle singular points* (*JASPs*), which present extreme at the joint angle traces. We assumed that the modeled patterns of *FMSPs* and *JASPs* may be applied for an analysis of the synergic interaction between the motor commands arriving at different muscles in arbitrary two-joint movements.

## Introduction

Currently, the existence of three interdependent types of synergies are discussed in studies devoted to the experimental analysis of human movements. Anatomical and neural factors combine to form coordinated joint movements, often referred to as kinematic synergies, i.e., simultaneous covariations in the relatively independent changes of the joint angles (Santello and Soechting, 2000). The presence of kinematic synergies has also been reported during manual exploration (Thakur et al., 2008) or during skilled movements, such as typing (Soechting and Flanders, 1997). Another type of synergistic effect, kinetic synergies, which are described by the covariation of forces (or torques), have also been observed when generating multi-finger forces. For example, during grasping tasks (Santello and Soechting, 2000) or during the forced interaction of various fingers (Grinyagin et al., 2005). Muscle synergies, which are based on the spatial and temporal coordination of multiple EMGs, have been observed during static hand postures (Weiss and Flanders, 2004; Castellini and van der Smagt, 2013) or during active force production between the muscles acting on the digits (Valero-Cuevas, 2000; Latash et al., 2007).

Despite the synergistic effects observed when predominantly studying complex purposive movements including many joints and muscle groups, it seems that analysis of the synergistic forms of muscle interaction may also be suitable for simple types of experimental movements, such as two-joint planar movements. Recent papers have attempted to find the elements of synergistic patterning even in highly simplified forms of real movements, which are largely used only in reduced experimental models of the synergies between muscles belonging exclusively to two neighboring joints (Abramovich et al., 2015; Hirai et al., 2015). We suppose that for such reduced models of movement, it would be preferred to use the term “quasi synergy” instead of the generally accepted synergy that is ordinarily only applied for real multi-joint movements. Our previous studies of two-joint movements produced under visual control (Abramovich et al., 2015, Tomiak et al., 2015) present examples of a quantitative approach to investigate both the EMGs coming to the muscles of the elbow and shoulder joints as well as the related patterns of their quasi synergistic effects. In the present paper, by using visual tracking for the test movements and basic methods for their analysis, we studied the patterns of EMG activity in the elbow and shoulder muscles for the circular movements of the right arm produced under action of loads directed tangentially to the movement trace; the analysis was widened by force moments computation. Based on a known place at the distal end of the two-joint system (hand coordinates) and the fixed position of the proximal (shoulder) joint, it is possible to evaluate the force moments and angles at both connected joints using a simple geometrical model of the system. Such a geometrical model of the two-joint movement allows the identification of important singular points along the movement trace where: (1) the force moments at the joints attain zero values (*force moment singular points—FMSPs*); (2) the movement direction at the joints is reversed (*angle singular points—JASPs*). One of the objectives of this study is to define the correspondence between the EMGs in different muscles and the biomechanical constraints imposed by the sets of the singular points. In general, an arbitrary movement trace may be presented by a set of circular segments of different diameters and positions. Therefore, one may assume that the proposed theoretical model will allow the production of a preliminary prediction of both the motor commands to the muscles during movement and their synergistic patterns.

*Hypothesis*. The motor commands to the muscles during two-joint circular movements are predominantly connected with changes in the force moments at the correspondent joints during movement; the commands are modulated in accordance with the eccentric or concentric character of the muscle contractions at the corresponding parts of the movement trajectory. The EMG patterns are largely defined by the location of *FMSPs* and *JASPs*; the exits of the averaged EMGs out of the trajectory fragments between neighboring *FMSPs* are likely connected with co-activation of the muscle-antagonists and/or with a more complex joint's geometry.

## Methods

### Experimental setup

**Figure 1:**
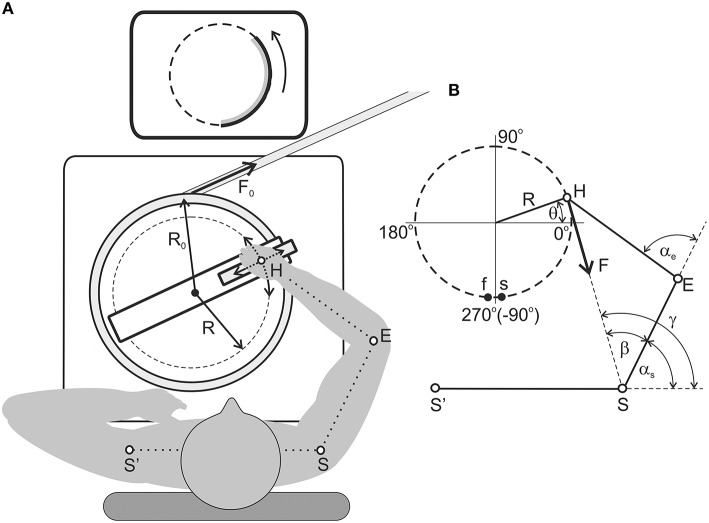
**Experimental setup. (A)** General appearance from a top view; a detailed explanation is given in the Methods section. The arrangement of the rubber band corresponds to the clockwise load; **(B)** Scheme used for the definition of the force moments and joint angles in the shoulder and elbow joints (Section Evaluation of the Movement-Dependent Changes of the Joint Angles and the Moments of the External Forces Acting around the Joints). The points *s* and *f* designate the *start* and *finish* of the counter-clockwise movements.

The experiments were conducted with six adult right-handed men between 21 and 27 years old (24.8 ± 3.5). All study procedures were in accordance with the ethical standards of the research committee of Bogomoletz Institute of Physiology, National Academy of Sciences, Kiev, Ukraine, and with the 1964 Helsinki declaration and its subsequent amendments or comparable ethical standards. Informed written consent was obtained from all individual participants included in the study. The experimental procedure lasted approximately 1 h. The mechanical component of the experimental setup is schematically presented in Figure 1. The setup is also described in details in our previous study (Abramovich et al., 2015). The mechanical system consisted of a rotating circular platform using ball bearing elements that was installed in a massive basement. A subject took a handle by the right hand, which was immobilized by a special cuff removing wrist movements; the handle was installed at a carriage moving in a radial direction of the rotating platform. Test movements were produced by the transition of the handle along the demanded circular trajectory, thus supporting a given invariable radial position; movements were fulfilled in the horizontal plane and passed approximately along the shoulder joint. Two signals from precise potentiometric sensors provided real-time information about the radial position of the handle (R) and turning angle (θ). These signals were used for on-line exhibition of the hand's position at the monitor screen as well as for off-line calculation of the joint angles and the force moments (see Section Evaluation of the Movement-Dependent Changes of the Joint Angles and the Moments of the External Forces Acting around the Joints). The radii of the turning platform (R_0_) and the movement circle (R) were 20 and 18 cm, respectively (Figure 1). The moment of external loading was provided by a stretched rubber band (6 m of length in the non-stretched state) connected to a string that was wrapped around the platform. The range of the tension changes during the single movement test was 1.3–2.0 H. By changing the wrapping direction of the string, it was possible to create external torque by turning the platform in clockwise (M_cw_) or counter-clockwise (M_ccw_) directions (Figure 1A). At each torque direction (M_cw_ or M_ccw_), a subject consecutively produced two tests that included full cycles of the slow circular movements in clockwise (L_cw_) or in counter-clockwise (L_ccw_) directions. Therefore, the following set of test movements were used: M_cw_ − L_cw_; M_cw_ − L_ccw_; M_ccw_ − L_cw_; M_ccw_ − L_ccw_. During the experiments, a subject sat in a chair in with a consistent chair-bottom position; his position was adjusted relative to the horizontal location of his entire right arm while his hand embraced the carriage handle. Special belts fixed the subject's trunk to the chair back to allow maximal fixation of the shoulder joint position. The movements were executed using a standard visual tracking method; a subject had to combine a beam projected on the monitor screen, which reflected the real position of his right hand, with another beam to produce a signal that in real-time was a circular trace. The position signal was computed on-line by using the θ and R signals. The test movements were produced with a constant velocity of 18°/s; the duration of the full-cycle movement was 45 s. In the case of the clockwise torques shown in Figure 1A, the first test movement began at point *s* and finished at point *f*. Then, after a 5 s rest period, a similar movement was initiated in the reverse direction (between *f* and *s* points). In the case of the oppositely directed torque (M_ccw_), the first movement test began at point *f* and finished at point *s*; the movement then elapsed in the reverse direction. Due to the constructive limitations of the experimental setup, points *s* and *f* were shifted by 4–6° from the line −90° (270°). Prior to each experiment, the distances between the centers of the shoulder and elbow joints and between the center of the elbow joint and the axis of the handle were measured for the following computation of the shoulder and elbow joint angles α_e_, α_s_ (Section Evaluation of the Movement-Dependent Changes of the Joint Angles and the Moments of the External Forces Acting around the Joints).

### Evaluation of the movement-dependent changes of the joint angles and the moments of the external forces acting around the joints

**Figure 2:**
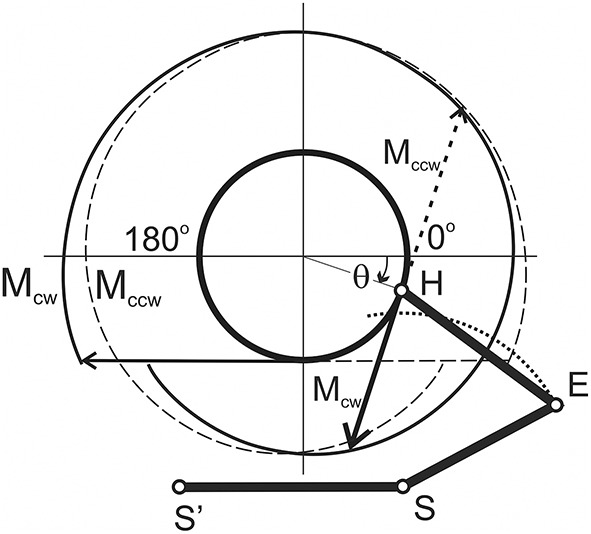
**Movement-dependent changes in the joint angles and the force moments acting at the elbow and shoulder joints during the tests**. The hodographs of the external torque vectors for the clockwise (M_cw_, solid line) and counter-clockwise (M_ccw_, dashed line) loadings. Their differences are connected with the length-dependent change in tension of the rubber band.

A simplified scheme of the arm segments and the movement trajectory is shown in Figures 1B, 2; it is used to determine the joint angles α_s_ and α_e_ and force moments M_s_ and M_e_ and their dependent relationship on the turning angle θ. Formally, the model of the arm includes two ideal ball-and-socket joints and linear arm segments, those lengths were defined for each subject before the experiment. At the initial stage, for both directions of loading (M_cw_, M_ccw_), there were defined changes in the moments of external load due to the length-dependent changes in the force of the rubber band:

(1) $$M\left(\theta \right)=\frac{(F_{2}-F_{1})R_{0}}{\left(\theta_{2}-\theta_{1}\right)}\;\cdot \;\left(\theta -\theta_{1}\right)+F_{1}R_{0},$$

where: θ_1, 2_, initial and final values of the turning angle; *R*_0_, radius of the platform, *R*, radius of the test movement, F_1, 2_, the measured values of the rubber band force at the boundaries of the movement diapason.

Cartesian projections of the force applied to the subject's hand at point H are as follows:

(2) $$\begin{array}{l} F_{x}=-\frac{M\left(\theta \right)}{R}\cdot cos\left(\theta \right);\;F_{y}=\frac{M\left(\theta \right)}{R}\cdot sin\left(\theta \right). \end{array}$$

The vector products define the force moments acting around the joints:

(3) $$\begin{array}{l} {\vec{M}}_{s}={\vec{r}}_{s}\times \vec{F};{\vec{M}}_{e}={\vec{r}}_{e}\times \vec{F} \end{array}$$

where subscripts s and e signify a relationship to the shoulder and elbow joints, respectively; ${\vec{r}}_{s}$- the vector directed from the shoulder joint to the wrist and the resulting SH distance, ${\vec{r}}_{e}$- the vector directed from the elbow joint to wrist and the resulting EH distance.

The vectors ${\vec{r}}_{s}$ and ${\vec{r}}_{e}$ are defined as follows:

(4) $$\begin{array}{l} {\vec{r}}_{s}=\left[\begin{array}{c} H_{x}-S_{x}\\ H_{y}-S_{y} \end{array}\right];{\vec{r}}_{e}=\left[\begin{array}{c} H_{x}-E_{x}\\ H_{y}-E_{y} \end{array}\right], \end{array}$$

where subscripts x and y signify projections of the corresponding axes (*S*, shoulder; *E*, elbow; *H*, handle) in Cartesian coordinates, respectively.

Similarly, the angles at the elbow and shoulder joints were defined (α_*e*_, α_*s*_). The distance from the shoulder joint to the handle is calculated as follows:

(5) $$\begin{array}{l} |{\vec{r}}_{s}|=\;\sqrt{{\left(H_{x}-S_{x}\right)}^{2}{+\left(H_{y}-S_{y}\right)}^{2}}. \end{array}$$

The “shoulder” and “forearm” lengths (*L*_*s*_ = SE*; L*_*e*_ = EH) are measured for every subject before an experiment. The angle is calculated according to the formula:

(6) $$\begin{array}{l} \gamma =atan\left(\frac{H_{y}-S_{y}}{H_{x}-S_{x}}\right). \end{array}$$

The joint angles α_*s*_, α_*a*_ are defined as follows:

(7) $$\begin{array}{l} \alpha_{s}=\gamma -\beta ;\;\alpha_{e}=\pi -\;acos\left(\frac{L_{e}^{2}+L_{s}^{2}-L^{2}}{2\cdot L_{e}^{2}\cdot L_{s}^{2}}\right) \end{array}$$

**Figure 3:**
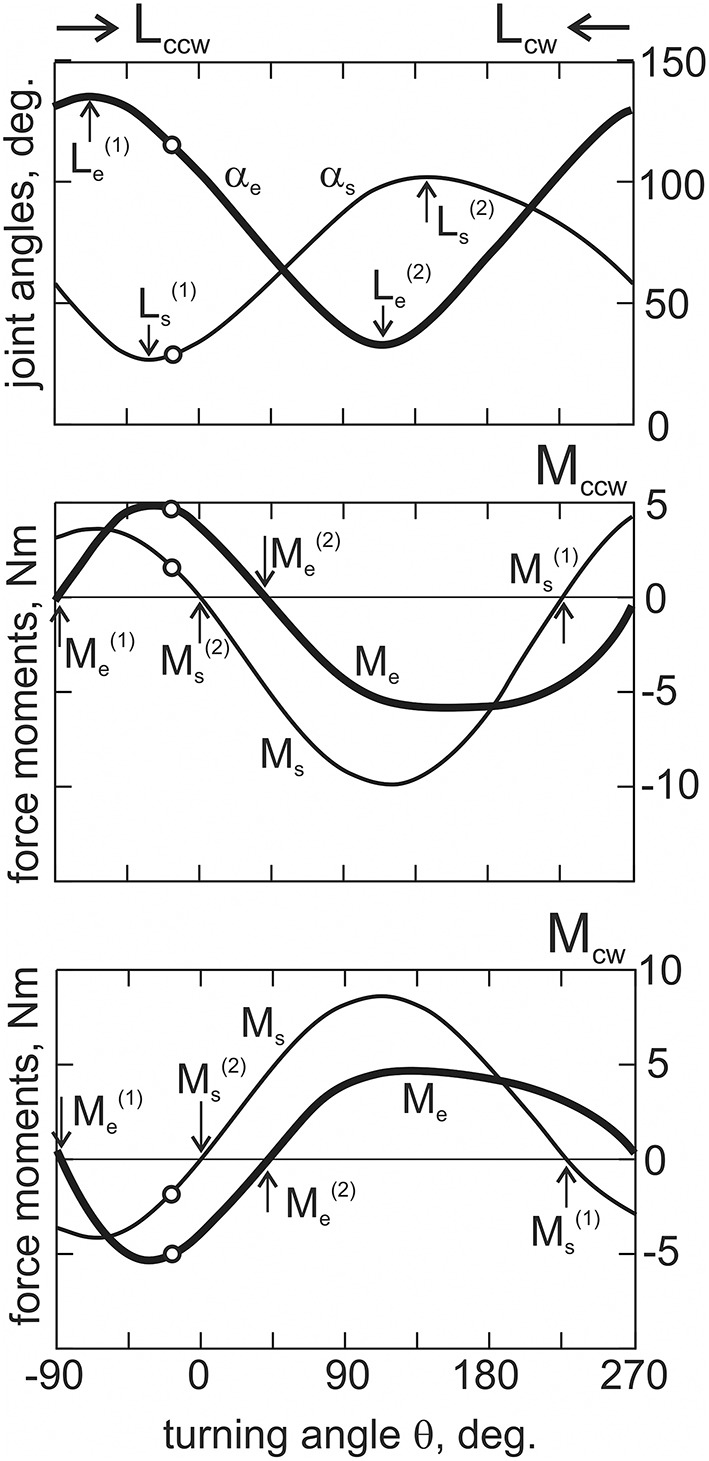
**The off-line computations of the joint angles and the force moments depending on the turning angle, θ**. Open circles on all graphs correspond to the arm position shown in Figure 2; vertical arrows designate the position and force singular points. The directions of the movement tests (L_cw_ and L_ccw_) are shown by horizontal arrows. The positive and negative directions of the force moments correspond to the loading of the flexor and extensor muscles, respectively. In this and the following Figures, subscripts signify: e, elbow; s, shoulder; cw, clockwise; ccw, counter-clockwise.

An example of the calculated changes of the joint angles and force moments and their dependency on the turning angle θ are shown in Figure 3; the results are obtained from an experiment that is further presented in **Figure 5**.

### EMG recording and off-line data processing

Surface EMGs were recorded using surface electrode pairs (Biopac System EL 503, USA; center to center distance 25 mm) that were fixed on the subject's right arm over the belly of the muscles. The electrodes were placed at the muscles under study in accordance with the schemas of Konrad (2006), which are widely used in the EMG studies. The activity was registered from eight muscles: *mm. pectoralis pars major, deltoideus pars scapularis, deltoideus pars clavicularis, biceps brachii caput longum, biceps brachii caput breve, brachioradialis, triceps brachii caput lateralis*, and *triceps brachii caput longum*. The recorded activity was amplified via a multichannel amplifier (16-channel Bioamplifier, CWE, Inc., PA 19003 USA) using a bandpass filter in the range of 10–5000 Hz. The EMGs together with the position signals θ and R were collected via a CED Power 1401 data acquisition system using the program Spike 2 (Cambridge Electronic Design, UK). The EMGs and the position signals were digitized at 10.0 and 2.0 kHz, respectively. Origin 8.5 (OriginLab Corporation, USA) and SPSS 17.0 (IBM Business Analytics software) were used for the off-line data analysis. The EMG records were full-wave rectified and filtered (Batterworth filter of 4th order, bandwidth 0–10 Hz) in an off-line regimen; this procedure introduced a phase lag with respect to the real changes in the EMG intensity near 130–150 ms; the angle errors for the used EMG—turning angle presentations did not exceed ± 2.7°. All tests were repeated 10 times to average the corresponding records. Prior to each experiment, we registered the MVC in each muscle undergoing study. For this purpose, the averaged EMG levels during steady-state maximal isometric contractions of the muscles when the shoulder and elbow angles were near 70 and 90°, respectively, were defined. Similarly, the minimal levels of EMG activity in fully relaxed muscles were evaluated. The averaged EMG activity registered in the main part of the experiments is shown in the percentage scales which ranged from the above-defined minimal levels of activity (0%) and the MVCs (100%).

### Statistical analysis

Statistical analysis was applied to the EMG activities of the muscles under study. In the framework of ANOVA analysis, the direction of external load (M), the direction of movement (L), and the zones of movement (Z) are considered as principal factors. The first two factors have two levels of change (M_ccw_ or M_cw_; L_ccw_ or L_cw_), whereas the third is defined by three levels (zones I, II, III and IV, V, VI for muscles of the elbow and shoulder joints, respectively). For each of the muscles, 10 trials were used in four combinations of the loading and movement directions (M_ccw_ − L_ccw_, M_ccw_ − L_cw_, M_cw_ − L_ccw_, M_cw_ − L_cw_); in each of the trials, the average EMG intensities are taken into account. The number of the averaged values of EMG that are included in the analysis was defined by the multiplication of the number of muscles (8), trails (10), directions of load (2), directions of movement (2), and zones of movement (3); therefore, this term equals 960 (8 × 10 × 2 × 2 × 3). *Post-hoc* analysis used Bonferroni pairwise comparison. Additionally, we calculated values of the observed power (π) for ANOVA results at 0.05 significance level. All statistical computations were performed by the programme SPSS Statistics 17.0(IBM, USA).

## Results

### The singular points and quasi synergy zones

**Figure 4:**
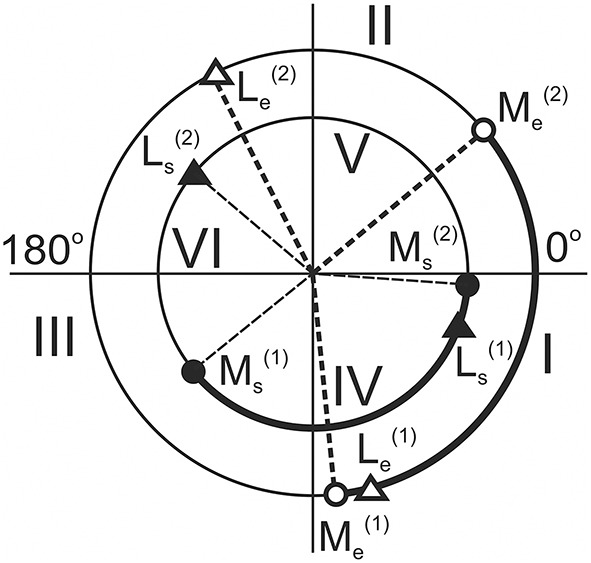
**The circular diagrams reproduce the angular positions of the singular points of the movement traces (in correspondence with Figure 3)**. Larger and smaller circles belong to the elbow and shoulder joints, respectively. Roman numerals note different zones at the movement traces with loading flexors: I (${\text{M}}_{\text{e}}^{\left(1\right)}$ – ${\text{M}}_{\text{e}}^{\left(2\right)}$) and IV (${\text{M}}_{\text{s}}^{\left(1\right)}$ – ${\text{M}}_{\text{s}}^{\left(2\right)}$) (marked by thick lines), and extensors: II + III (${\text{M}}_{\text{e}}^{\left(2\right)}$ − ${\text{L}}_{\text{e}}^{\left(2\right)}$ − ${\text{M}}_{\text{e}}^{\left(1\right)}$) and V + VI (${\text{M}}_{\text{s}}^{\left(2\right)}$ – ${\text{L}}_{\text{s}}^{\left(2\right)}$ – ${\text{M}}_{\text{s}}^{\left(1\right)}$) (thin lines).

By using the procedure described in Section Evaluation of the Movement-Dependent Changes of the Joint Angles and the Moments of the External Forces Acting around the Joints, it is possible to define changes in both the joint angles and the forces acting on the muscles during movement (Figures 2, 3). These dependencies define the position at the movement traces where the external forces acting on the different muscle groups change their direction, *FMSPs*: ${\text{M}}_{\text{s}}^{\left(1,2\right)}$ and ${\text{M}}_{\text{e}}^{\left(1,2\right)}$, as well as where the muscles pass from lengthening to shortening and vice versa, *JASPs*: ${\text{L}}_{\text{s}}^{\left(1,2\right)}$ and ${\text{L}}_{\text{e}}^{\left(1,2\right)}$. The singular points are easily defined at the reconstructed dependencies of the corresponding mechanical parameters of movement on the turning angle (Figure 3). For each of the joints, the direction of the change in muscle length is altered twice during a full cycle of movement: *JASPs* ${\text{L}}_{\text{s}}^{\left(1,2\right)}$ and ${\text{L}}_{\text{e}}^{\left(1,2\right)}$ (upper panel in Figure 3); *FMSPs* ${\text{M}}_{\text{s}}^{\left(1,2\right)}$ and ${\text{M}}_{\text{e}}^{\left(1,2\right)}$ coincide with the sign reversing position at the moment curves (seconds and third panels in Figure 3). In spite of some differences in the form of the force moment curves for different loading directions, the positions of ${\text{M}}_{\text{s}}^{\left(1,2\right)}$ and ${\text{M}}_{\text{e}}^{\left(1,2\right)}$ are coincident for both. *FMSPs* and *JASPs* can be marked at the movement traces; for convenience, the points belonging to different joints may be shown schematically at separate concentric circles (Figure 4). The singular points divide the movement trajectories on the zones with a different muscular mechanical state. In general, when the singular points belonging to different joints that do not coincide with each other, they will divide both circles into four sectors. However, the considerably close locations of the *JASPs* and *FMSPs*, i.e., ${\text{L}}_{\text{e}}^{\left(1\right)}$, ${\text{M}}_{\text{e}}^{\left(1\right)}$ (elbow), and ${\text{L}}_{\text{s}}^{\left(1\right)}$, ${\text{M}}_{\text{s}}^{\left(2\right)}$ (shoulder), allow the use of only three sectors for the purposes of the following analysis. During the first step, one can neglect the effects connected with *JASPs* ${\text{L}}_{\text{e}}^{\left(1\right)}$ and ${\text{L}}_{\text{s}}^{\left(1\right)}$, which are too closely positioned to their corresponding *FMSPs*. Thus, we will further use three-zone partitioning for the test movements: I – III for the elbow and IV – VI for the shoulder joints. For the two possible directions of the external load (M_ccw_; M_cw_), the loads applied to each of the joints consist of two waves in the opposite direction; one wave loads flexors, while another acts against extensors. Alterations in the load direction lead to inversion of the moment curves, whereas the positions of the respective *FMSPs* remain unchanged. Within the entire group of subjects, *FMSPs* points are closely disposed to each other; their positions are 85.54 ± 2.9 (${\text{M}}_{\text{e}}^{\left(1\right)}$), 36.77 ± 3.3 (${\text{M}}_{\text{e}}^{\left(2\right)}$), 223.21± 5.6 (${\text{M}}_{\text{s}}^{\left(1\right)}$), and −2.37±1.6 (${\text{M}}_{\text{s}}^{\left(2\right)}$) degrees (*mean* ± *m.s.e*.).

Due to the unequal durations of the loading waves with respect to the full duration of the movement cycle, the reverse of the external load alters the ratio between the durations of the loading cycles applied to the muscle-antagonists (Figures 3, 4). During the action of the counter-clockwise loads (M_ccw_), the flexor muscles are loading in sectors I (elbow) and IV (shoulder), which are marked by thick lines on the circular diagrams. In contrast, the extensor muscles obtain loading in sectors II+III (elbow) and V + VI (shoulder), as shown by thin lines. Sectors I and IV, where the load is applied to the flexors during M_ccw_, occupy relatively smaller parts of the full circles (Δα_I_ < 180°; Δα_IV_ < 180°), whereas sectors where the external load acts on the extensors are somewhat bigger (Δα_II+II_ > 180°; Δα_V+VI_> 180°). During application of M_cw_, the loading order is swapped between antagonists and the extensors become loaded in sectors I (elbow) and IV (shoulder). Due to the constant locations of *FMSPs* along the movement trajectory, all sectors are fixed.

### EMGs in various combinations of the movement and load directions

**Figure 5:**
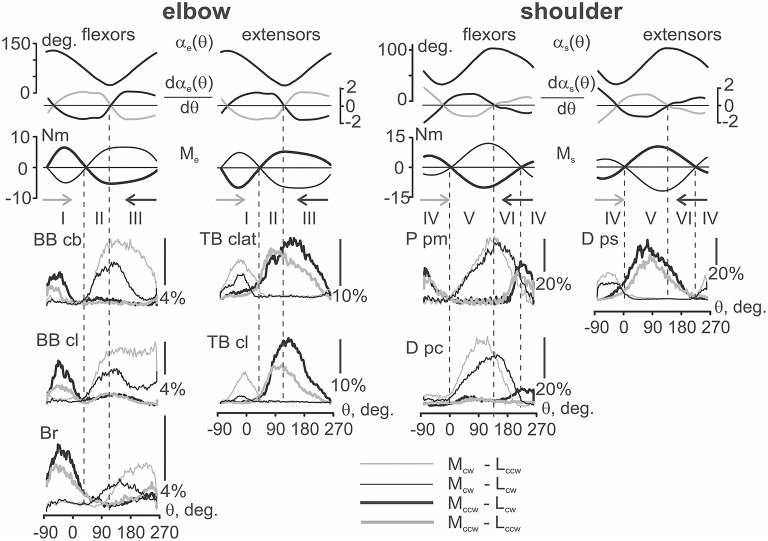
**The mechanical parameters of movement and the averaged EMGs dependent on the turning angle for four different combinations of the load and movement directions**. The first three rows present the joint angles. Their first derivatives are defined with respect to the turning angles for movements in clockwise (black) or counter-clockwise (gray) directions. The force moments at the joints during action of the clockwise (thin) and counter-clockwise (thick) loads; the colors and line thickness are used to distinguish proper EMG records. The derivative traces are used only as indicators of the direction in the length changes of the corresponding muscles; the traces have been inverted for flexors to correctly note the directions of their shortening (negativity of the curves) or lengthening (positivity). The force moment traces for the extensors are also inverted compared with the flexors; in both the flexors and extensors, only the positive parts of the moments should be taken into account for the analysis of their loading. Vertical dashed lines mark the movement zones determined in Figure 4. The EMG calibration is presented in percentage of MVC; the abbreviations for the muscles are presented at the beginning of the paper.

An example of a typical experiment that records the averaged EMGs in four combinations of the external torque and movement directions is shown in Figure 5. The standard set of mechanical components is shown above in Figure 3, and here we also added the first derivatives of the joint angle changes. In each of the joints, the EMGs recorded from the flexor and extensor muscles are tightly connected with mechanical components of movement, which correlate with the positive parts of the loading waves applied directly to the muscles. First, one can consider only reactions based on a change in the loading directions, thus comparing pairs of lines of the same thickness in Figure 5. The EMGs presented by thick lines (M_ccw_ loads) are localized predominantly within the I (elbow) and IV (shoulder) zones for flexors, and within II+III (elbow) and V+VI (shoulder) zones for extensors. On the contrary, the EMGs shown by thin lines (M_cw_ loads) are localized predominantly within the II+III (elbow) and V+VI (shoulder) zones for flexors, and within the I (elbow) and IV (shoulder) zones for extensors. By neglecting some exits of the EMG intensities into neighboring zones of movements with zero load, it is possible to assure that the recorded EMGs from the muscles are correlated with the phases of their loading. Such a correlation is better observed in the elbow muscles, whereas more significant deflections and variability are present in the shoulder muscles.

For the same load directions, it is also possible to observe differences in the EMGs recorded at different movement directions. Most of the EMG intensities are usually observed for one of the movement directions (L_ccw_ or L_cw_); the observed ratio between the EMG intensities is reversed with a change in the load direction (M_ccw_ or M_cw_). This is clearly seen in Figure 5, where for the pairs of the EMGs registered during the action of M_ccw_ loads (black and gray thick lines); the records obtained during clockwise movements (black) are generally above the corresponding counter-clockwise reactions (red). In contrast, within pairs of the thin lines presenting the EMGs during M_cw_ loads, the black lines (L_cw_) are mostly below the gray (L_ccw_) lines. It is interesting that this order is observed at both joints and is valid for both flexor and extensor muscles.

The movement-dependent differences between EMGs are likely simpler and more predictable in zones I (elbow) and IV (shoulder), where, as it was noted earlier, the *JASPs* are placed closely to the corresponding *FMSPs*. It is very likely that the action of the movement-dependent effects cannot be noticeably displayed in these small areas of the movement traces, which simplify the definition of the relationship between the EMG intensities in the above movement zones. An actively shortening muscle (*concentric contraction*) generates more intensive EMGs, and, on the contrary, when a muscle is lengthening by the exceeding external load (*eccentric contraction*) it generates less activity. First, this relates to reactions of the elbow flexors and extensors in zone I; a reverse order in the positioning of the different color curves may be trivially explained in this case as well.

A more complex situation is present for wider sectors of loading, each of which are divided by the proper internal *JASPs* into two zones: II + III (elbow) and V + VI (shoulder). In the vicinity of these points, several muscles demonstrate obvious direction-dependent cross-overs of the EMG traces. For the elbow flexors, such reactions can be noted only in *Br*; in this case, the thin black line (M_cw_ − L_cw_ test) goes above the thin gray line (M_cw_ − L_ccw_) in zone II. Then, after crossing these lines near the boundary of zones II and III, the black line moves down and falls below the gray line. Similar cross-overs of the EMG traces in opposite movement directions are seen in the elbow extensor *TB clat* and in the shoulder flexors *P pm* and *D pc*. In all of these cases, the actively contracting muscles generate greater EMG intensities, and a change in movement direction at the corresponding PSPs evokes a predictable alteration in the ratio of the activation intensities in these muscles. A significant divergence of the EMG curves in wider movement-related zones (III and V) can lead to violation of the crossing-over rule during transition to narrower parts (II and VI); it seems that in these cases, there is insufficient time for the development of the corresponding reaction. Such a behavior may be observed in the EMGs recorded from *BB cb, BB cl, TB cl*, and *D ps*, when a noticeable rise in the EMG intensities during active shortening in wider zones III and V does not permit the necessary activation intensity by the muscles in the narrower zones II and VI.

### Statistical analysis of the EMG intensities in different parts of the movement traces

Strong dependency of the EMG intensities on both the load and movement directions, as well as clear differences between the reactions in different zones of the movement trajectories, create the prerequisites for the use of ANOVA to quantitatively characterize the variety of the motor commands (Table 1). In the framework of this analysis, the direction of external load (M), the direction of movement (L), and the zones of movement (Z) are considered principal factors. The first two factors have two levels of change (M_ccw_ or M_cw_; L_ccw_ or L_cw_), whereas the third is defined by three levels (zones I, II, III and IV, V, VI for muscles of the elbow and shoulder joints, respectively).

**Table 1:** **Results of the ANOVA analysis of the test movements presented in Figure 5**.

	**M**		**L**		**Z**		M^*^L		M^*^Z		L^*^Z		M^*^L^*^Z	
**Muscle**	***F***	**Sig**.	***F***	**Sig**.	***F***	**Sig**.	***F***	**Sig**.	***F***	**Sig**.	***F***	**Sig**.	***F***	**Sig**.
*BB cb*	399.57	0.000	33.80	0.000	78.26	0.000	94.54	0.000	181.94	0.000	38.10	0.000	18.63	0.000
*BB cl*	194.05	0.000	25.04	0.000	44.65	0.000	80.40	0.000	178.93	0.000	31.18	0.000	13.67	0.000
*Br*	38.91	0.000	**0.01**	**0.903**	168.96	0.000	17.48	0.000	512.13	0.000	64.24	0.000	26.08	0.000
*TB clat*	1308.82	0.000	**0.17**	**0.674**	66.02	0.000	123.46	0.000	1039.22	0.000	88.69	0.000	72.11	0.000
*TB cl*	751.58	0.000	18.99	0.000	115.59	0.000	160.77	0.000	475.23	0.000	84.87	0.000	21.52	0.000
*P m*	673.45	0.000	**2.94**	**0.089**	266.94	0.000	**0.30**	**0.582**	420.08	0.000	5.29	0.006	6.61	0.002
*D pc*	216.97	0.000	**0.08**	**0.777**	63.85	0.000	**2.74**	**0.100**	51.12	0.000	11.98	0.000	6.33	0.003
*D ps*	986.19	0.000	14.95	0.000	281.13	0.000	94.67	0.000	926.42	0.000	44.46	0.000	18.08	0.000

*Definition of factors: M, factor of the loading direction (two levels: M_ccw_; M_cw_); L, factor of the movement direction (two levels: L_ccw_; L_cw_); Z, factor of the zone (three levels: zones I, II, III for the elbow joint; zones IV, V, VI for the shoulder joint); asterisks are used to describe the interactions of the factors. Boundaries of the zones are defined in accordance with Figures 4, 5. The gray colored cells mark the absence of statistical significance for a given parameter, i.e., p > 0.05. Additionally, there was defined parameter of the observed power (π); in the cells marked by bold font π < 0.4, while at the rest of the cells this parameter exceeded 0.8*.

As can be seen from Table 1, the average EMG intensities in the muscles are strictly dependent on the experimental conditions. In agreement with the above qualitative description of the experimental records in Figure 5, a strong dependency of the EMG levels on the loading directions (column M in Table 1) can be noted. On the other hand, the direction of movement (column L) provides statistically significant action on the muscle reactions in only half of the cases, which is likely connected with a proximity to the integral parameters of the EMG responses for oppositely directed movements, as can be seen, for example, in reactions *Br* and *P pm* (Figure 5). A lack of determinacy in the influence of the movement direction factor on the EMG activities may constitute a reason for the absence of statistical significance for the action of the combination of factors of the load and movement directions for the *P pm* and *D pc* muscles (M^*^L column in Table 1). In summary, we would like to stress that strong influences of the M and Z factors were observed in all muscles, whereas the Z factor seems to provide statistical significance for the combination of the factors: M^*^Z, L^*^Z, M^*^L^*^Z. The last conclusion is in agreement with the absence of significance for the M^*^L combination in a part of the muscles (Table 1).

**Figure 6:**
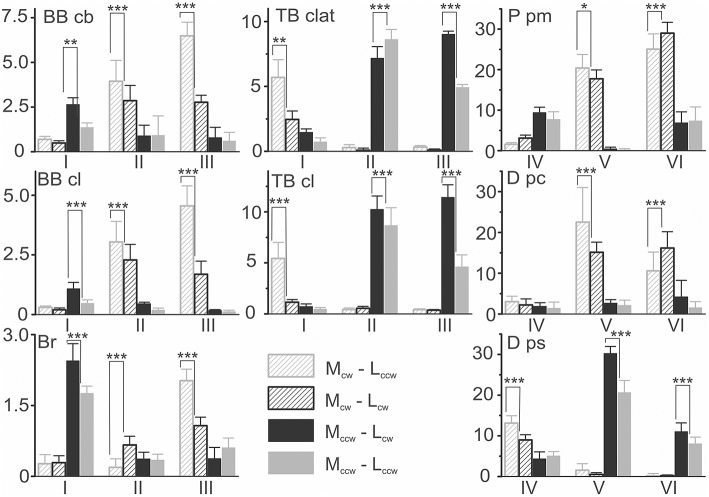
**Statistical analysis of the EMG intensities within different movement zones for the experiment presented in Figure 5**. The EMG parameters in percent of MVC (mean ± m.s.e.) for four combinations of external torque (M_cw_; M_ccw_) and the movement directions (L_cw_; L_ccw_) are compared. The results of the Bonferroni post-hoc analysis of pairwise comparisons for the movement-dependent differences in the EMG intensities within the same zones are schematically indicated by asterisks (^*^p < 0.05; ^**^p < 0.01; ^***^p < 0.005).

The presence of a strong dependency of the EMG intensities in different muscles on both the load and movement directions, as well as clear differences in the reactions in different zones, allowed us to apply *post-hoc* Bonferroni pairwise comparisons to reveal probable differences between various components of the motor commands. This analysis is presented in Figure 6; it quantitatively supports the inferences made above for consideration of the averaged EMGs at four different combinations in the directions of the external load and movement. For example, it statistically supports the existence of a difference between reactions *Br* and the heads of the biceps, *BB cb* and *BB cl*, whose responses were quite similar. It also implicates a larger relative weight of the *Br* reactions for the M_ccw_ loads (zone I, black and gray bars) compared with those of M_cw_ (zones II, III, dashed black and gray bars). It seems to also be important that a direction-dependent reversal of the EMG intensities in zones II and III is observed only in *Br*; it was not present in both biceps heads. It may be noted that that such a reversal is statistically significant only in *TB clat* and is absent in *TB cl*. In the shoulder muscles, if a comparison of their high-amplitude reactions in the V and VI zones is made, the reversal is present only in flexors *P pm* and *D pc*, whereas the extensor *D ps* does not demonstrate this property.

### Averaging of the EMG reactions in the group of subjects

**Figure 7:**
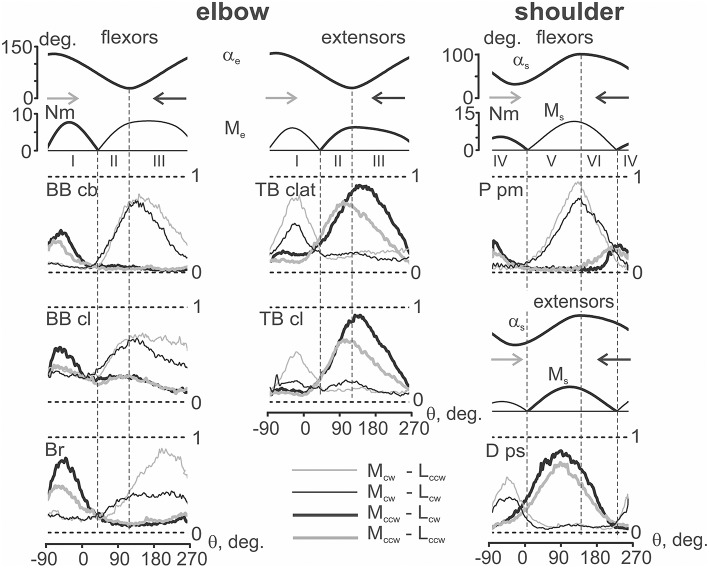
**Averaging of the EMG patterns registered during standard movement tests (as shown in Figure 3) in the group of six subjects**. Before the averaging, the corresponding EMG records were normalized in each of the subjects with respect to their maximal values achieved in a given group of four tests. The EMGs are marked in a similar way as in Figure 3; horizontal dashed lines note the normalization scale (0, 1). The joint angles and moments have been averaged without normalization; only the positive parts of the moments are shown.

The EMG reactions were similar for the entire group of subjects, coinciding with the results described above for a single person. We have summarized the results by the special procedure of group averaging the parameters that were studied (Figure 7). The unified tests in these experiments allowed us to apply standard averaging for the recorded mechanical parameters; to compare the EMGs registered in various subjects, preliminary normalization was used. First, within the group of the four standard tests, there were defined minimal and maximal values of the averaged EMGs for each of the muscles under study. Seconds, the scales of the EMG records in these groups were normalized with respect to the above defined maximum and minimum, which were accepted as one and zero, respectively. After normalization, the EMG traces belonging to the proper combination of load and movement directions were averaged within the group of subjects.

The group-averaged EMG records presented in Figure 7 mainly resemble the corresponding reactions registered in a single subject (Figure 5). Both the general appearance of the reactions and their variability for different loads and movement directions are quite similar. While comparing the same loading directions, a clear divergence between the EMG traces belonging to opposite movement directions can be noted. Moreover, a tendency for more evident differences between “*eccentric*” and “*concentric”* EMG traces in zones II (elbow) and VI (shoulder) appeared, although this was not seen in all cases (for example, in *D ps*). At the same time, for a given loading direction, a movement-dependent divergence of the EMG traces is better observed in zones III (elbow) and V (shoulder).

The main inference that could be made from the comparisons of the motor commands to muscles in different combinations of load and movement directions is as follows. When the movement and load are directed in opposition to each other, i.e., in combinations M_cw_ − L_ccw_ for flexors and M_ccw_ − L_cw_ for extensors, the corresponding muscles are actively shortening and generate more intensive EMGs (*concentric contractions*). On the contrary, during coinciding combinations of directions, M_cw_ − L_cw_ for flexors and M_ccw_ − L_ccw_ for extensors, the test movements are accompanied by muscle lengthening when the muscle is less active (*eccentric contractions*). This scheme explains the observed differences in the EMG traces in the flexors and extensors.

Patterns of co-contraction of the antagonist muscles during fulfillment of the circular two-joint movements mainly coincide with the individual and group averaging (compare Figures 3, 5). The co-contractions are predominantly displayed as exits of activity in a given muscle out of its loading zone. For example, such an activity can be noted for the reactions of *TB clat* and *TB cl* in zone I, *P pm* in zone VI, and *D ps* in zone IV (Figure 7, thick lines, M_ccw_ loads). It is interesting that the co-activation patterns are quite stable and are almost independent on the movement direction.

## Discussion

By using the present experimental model, we attempted to find a simplified scheme for the quasi synergistic effects in circular movements, which may be presented by the pure formal interaction between loading and the activation patterns of the muscles belonging to different joints. The *force quasi synergy* was defined in the framework of the simplified arm model that includes two ideal ball-and-socket joints and linear arm segments whose lengths were defined with a sufficient precision for each subject before the experiment. The model allows the production of off-line computation of the joint angles α_s_ and α_e_ and the force moments M_s_ and M_e_ depending on the turning angle θ, which is defined as the current hand position of the circle during movement. The *force quasi synergy* is largely defined by the set of four *FMSPs* (two for each joint), in which the force moments at the joints change their signs. During conditions of fixed placement of the shoulder joint, invariable lengths of the arm segments and constant parameters of the movement trace (position of the center and radius of the circle), the arrangement of *FMSPs* along the movement path are invariable for both clockwise and counter-clockwise directions of external load. For each of the joints, two *FMSPs* divide the movement trace into two unequal segments where the oppositely directed force moments are applied by turning to the antagonists. At M_ccw_ loads, shorter waves of loading act on flexors at both joints, while longer waves act on extensors; at M_cw_ loads, this order is reversed, so the longer (shorter) waves of load are applied to the flexors (extensors). There exists four combinations during simultaneous loading of the antagonistic muscles belonging to different joints: f_s_−f_e_, e_s_−e_e_, f_s_−e_e_, e_s_−f_e_ (f, flexors; e, extensors; indices: s, shoulder; e, elbow) (Figure 4). Therefore, for a given movement program, four types of the *force quasi synergy* may be distinguished.

In contrast to the *force quasi synergy* effects that had been modeled in the present study, the *activation quasi synergy* effects are registered experimentally. One can see that the positioning of the EMG waves along movement traces in the muscles of both joints largely correspond with the modeled moment waves. Therefore, the patterns of the real *activation quasi synergy* are quite similar with the theoretically defined *force quasi synergy*. However, at least two principal distinctions can be noted. First, the EMG activities of the muscles are not completely concentrated within zones restricted by *FMSPs*; their exits out of the boundaries are often encountered. Seconds, the activities of all muscles are dependent on the movement direction, which is obviously connected with differences in the eccentric and concentric contractions. At the same time, these differences in the EMG intensities are statistically significant only in the wider parts of zones III (elbow) and V (shoulder) (Table 1, Figure 6).

The exit of the EMG activity in the muscles away from their zones of loading may be connected with a more complicated arrangement of the joints compared with the assumed pivotal form. Analysis of a complex geometry of the rotational movements in the shoulder joint can be found in a previous reference (Hill et al., 2008) that presents the models with two and three degrees of freedom. Elbow joint biomechanics are highly intricate; they were recently considered a constellation of three interactive joints (Bryce and Armstrong, 2008). It was noted in the previous paper that the axis of elbow joint rotation can change its position and direction even in the absence of movement in the shoulder joint. It seems that complex mechanical systems such as the elbow and shoulder joints could possess the elements of indeterminacy during changes in the moment sign during movement. On the other hand, the reactions of different muscles in the same joint also varied. For example, exits of activity were noticed in *TB clat* and *Tr cl*, which demonstrate a spreading of the EMG intensity from zone II to zone I, whereas the activities of both biceps heads are more clearly restricted by their zones of loading (Figures 3, 5). On the contrary, in the shoulder joint, activity exits are observed in both flexors and extensors.

The EMG reactions in the muscles depend on the directions of both the external load and movement. As can be seen in the EMG records from *BB cb* in Figure 7, assisting action of the external loads almost completely removes the muscle activity (zones II and III for M_ccw_, and I for M_cw_ loads). In these sectors of movement, the EMG reactions are quite small, and their dependency on the movement direction is insignificant. Thus, the movement-dependent effects can appear only during muscle loading (zones I for M_ccw_, and II and III for M_cw_), where positioning effects are related to differences between concentric and eccentric types of contraction in the corresponding muscles. It is likely that a strong action of force effects on the EMGs leads to close interdependence between *force* and *activation quasi synergies*.

It should be noted that a division of the muscles with respect to their isolated movements around a given joint is oversimplified. This assumption may be true only for mono-articular muscles, such as *BB cl, Br, D ps*, and *P pm*. On the contrary, *BB cb* and *TB cl* are bi-articular muscles, which primarily provide movements around the elbow joint (Van Bolhuis et al., 1998). At the same time, excessive functional detail for the muscles participating in a given movement program can be unnecessary to analyze the possible effects of the *quasi synergy*, which are a reflection of the task goals and constraints rather than the fine details of the underlying hardware (Chhabra and Jacobs, 2006).

In the present study, the two-joint movements provide curvilinear trajectories of the distal parts of the arm in conditions of action of continuously changing loads. In spite of these movements are produced under visual guidance, it is quite clear that CNS may use various kinds of proprioceptive information to regulate action of the descending motor commands. The results of the study allows to separate two most important elements in this information.

First, it concerns of the sensory signals allowing to judge about presence or absence of loading in the muscles-antagonists at the both joints. One can speculate that any crossing of the *FMSPs*, in which the force moments alter sign, would evoke correspondent change of the afferent feedback signals, informing the motor system about necessity to redirect the descending activity between groups of the antagonistic muscles of the given joint. As a result, the descending activation diminishes in the previously contracted muscles, and rises in their antagonists. The Golgi tendon organs may present a main source for such the force information signals, although other afferent systems can be also involved as well (Proske et al., 2012).

Seconds, an important switching over between different kinds of the peripheral sensory flows may be connected with *JASPs*, indicating about transitions between lengthening and shortening of the muscles. Under artificial conditions of constant efferent activation, the muscle contraction dynamics is known to be crucially dependent on the movement direction; differences between eccentric and concentric types in the muscle activities are usually treaded as the length-tension hysteresis (Kostyukov, 1998). When the muscles are incorporated into the functioning spinal cord circuitries, such as the stretch reflex, the correspondent hysteresis loops become wider (Kostyukov, 1989), and the hysteresis amplification is mainly connected with direction-dependent asymmetries in activity of the muscle spindle afferents (Kostyukov and Cherkassky, 1997). Therefore, one can expect that the muscle spindle afferents may create essential involvement in forming direction-dependent differences between correspondent EMG traces registered in the present study.

Special areas can be separated at the movement trajectories, in which the programs of co-contraction of the muscle-antagonists predominate. The “co-contracting” muscles do not obtain direct external loading in such areas, therefore these muscles oppose the contraction of the loaded antagonists. These areas are localized strictly near zones where the muscles have been loaded earlier or will get loaded later, depending on the movement direction. Recently, the movement dynamics under these basic patterns of activation were studied in an experimental model of two antagonistic muscles (Gorkovenko et al., 2012). It was demonstrated that the co-contraction patterns can distinctly reduce the undesirable hysteresis after-effects, such as the ongoing residual movements at the apexes of activity. Thus, the co-activation of the antagonistic muscles may likely reduce the uncertainty effects in the motor control system, which are connected with the after-effects of muscle hysteresis (Kostyukov, 1998; Gorkovenko et al., 2012). Behavioral studies of postural tasks have demonstrated that subjects use muscle co-contraction as a strategy to stabilize limb joints in the presence of external loads (Kearney and Hunter, 1990; Milner and Cloutier, 1998). Humans are also able to independently modulate the relative balance of co-contraction and limb stiffness in different spatial directions (Burdet et al., 2001) and at different joints (Gribble and Ostry, 1998). It has been suggested that the CNS may use co-contraction as a strategy to facilitate accuracy of limb movement (Gribble and Ostry, 1998; Gribble et al., 2003).

Real multi-joint movement trajectories of movement in space inevitably contain linear and curvilinear elements, which could largely be approximated by separate parts of the circular traces of various diameters and space orientations. In many cases, for the analysis of muscle synergies it may be sufficient to define only the positions of singular points at the movement traces without precise computation of the force moment and joint angle traces. Therefore, a simplified method which would allow the determination of the *FMSPs* and *JASPs* in the multi-joint movements may be rather effective for the analysis of their synergies.

### Limitations of the present study

Limitations of the study are partly connected with a specific design of the setup, which, in particular, does not provide a constancy of the external force moments during test movements. The time lags between the smoothed EMG records and the real changes of EMG intensity were not compensated for, and the errors for the EMG—turning angle dependencies could be as much as 2.7° for the used velocity of movement. This study is considered as preliminary; the main part of the data analysis is connected with the statistical procedures applied to the EMGs recorded for 10 time repetitions of each of four standard movement tests in one of the subjects. At the same time, the method of averaging of the correspondent normalized averaged EMGs registered in six subjects allows to make conclusion about qualitative similarity of the observed EMG patterns in different subjects.

## Conclusions

The general features of the motor commands to the muscles in two-joint circular movements can be predicted with a simplified geometrical model of the arm. The motor commands are predominantly connected with changes in the force moments at the corresponding joints during movement and are modulated in accordance with the eccentric or concentric character of the muscle contractions at the corresponding parts of the movement trajectory. The EMG patterns are largely defined by the location of *FMSPs* and *JASPs*; the exits of the averaged EMGs out of the trajectory fragments between neighboring *FMSPs* may likely be connected with the co-activation of the muscle-antagonists and/or with a more complex joints' geometry.

## Author contributions

TT, discussion of experimental data; TA, explanation of the experimental data; AG, statistical analysis; IV, discussion of experimental data; VM, explanation of the experimental data; MD, discussion of experimental data; AK, modeling and discussion of experimental data.

### Conflict of interest statement

The authors declare that the research was conducted in the absence of any commercial or financial relationships that could be construed as a potential conflict of interest.

## Abbreviations

*BB cb*: m.biceps brachii caput breve
*BB cl*: m. biceps brachii caput longum
*Br*: m. brachioradialis
*TB clat*: m. triceps brachii caput lateralis
*TB cl*: m. triceps brachii caput longum
*P pm*: m. pectoralis pars major
*D ps*.: m. deltoideus pars scapularis
*D pc*.: m. deltoideus pars clavicularis
EMG: electromyogram
CNS: central nervous system
MVC: maximal voluntary contraction
*FMSP*: force moment singular point
*JASP*: joint angle singular point.
